# Multi-Walled Carbon Nanotube Growth on Fe/Al-Coated Thermally Stable Glass Substrates with Relevance to Field Emission

**DOI:** 10.3390/ma18174028

**Published:** 2025-08-28

**Authors:** Yung-Jui Huang, Guang-Yi Zeng, Lei Hu, Kuei-Yi Lee, Huan-Chun Wang, Pao-Hung Lin

**Affiliations:** 1 Department of Electronic and Computer Engineering, National Taiwan University of Science and Technology, Taipei 106335, Taiwan; 2 Graduate Institute of Electro-Optical Engineering, National Taiwan University of Science and Technology, Taipei 106335, Taiwan

**Keywords:** multi-walled carbon nanotube, glass substrate, buffer layer, chemical vapor deposition, field emission

## Abstract

The integration of vertically aligned carbon nanotubes (CNTs) onto glass substrates is a critical step toward realizing transparent and microfabrication-compatible electronic devices. The direct synthesis of patterned vertically aligned multi-walled CNTs (MWCNTs) on glass substrates using chemical vapor deposition (CVD) is demonstrated. Photolithographic patterning was employed prior to CNT growth to define the spatial geometry of the vertically aligned MWCNTs, enabling precise control over the emitter layout. A key factor influencing CNT morphology was found to be the thickness of the Al buffer layer. Among the tested thicknesses, an aluminum (Al) buffer layer with a thickness of 5 nm yielded optimal results. This configuration facilitates the growth of highly aligned MWCNTs with an average length of approximately 7 μm and a number density of about 10^9^ cm^−2^. The patterned MWCNTs exhibit excellent vertical alignment and well-defined hexagonal geometries consistent with photolithographic designs. Field emission measurements further validate the material quality, with patterned vertically aligned MWCNTs demonstrating uniform emission and good temporal stability. These results establish a practical and scalable approach for growing patterned vertically aligned MWCNTs directly on thermally stable glass substrates, offering a promising platform for transparent field emission technologies and CNT-based microsystems.

## 1. Introduction

Carbon nanotubes (CNTs) [1] have garnered significant attention as nanoscale materials for electronic, sensing, and energy-related applications due to their unique structural, electrical, and mechanical properties [2,3,4]. Among various configurations, vertically aligned CNTs are particularly attractive for device integration because their highly ordered structure enables anisotropic electrical and thermal transport, uniform surface morphology, and consistent material performance [5,6]. Precise control over the spatial arrangement and alignment of CNTs is critical for their effective integration into microsystems. The patterned vertically aligned CNTs, where the growth area and geometry are predefined using standard photolithography, offer a promising route toward scalable and application-specific fabrication of CNT-based components [7,8,9].

Several studies [10,11,12,13] have demonstrated that the field emission properties of CNT arrays are strongly influenced by their morphology, crystallinity, and substrate engineering. For instance, well-aligned and discrete CNTs have been shown to exhibit lower turn-on fields and higher emission uniformity [10]. The introduction of a titanium nitride (TiN) interlayer can reduce the turn-on field by up to 59% without altering CNT morphology, which is attributed to enhanced electron transport and heat dissipation [11]. The use of a W–Co bimetallic catalyst has been found to promote high-crystallinity CNT growth, resulting in improved emission stability and uniformity [12]. Furthermore, nanoimprint lithography enables precise definition of catalyst patterns down to sub-100 nm scales, facilitating controlled synthesis of high-aspect-ratio CNT arrays with tailored geometries [13]. These advancements collectively underscore the importance of integrating material engineering, catalyst design, and nanoscale patterning in CNT device fabrication.

Despite significant advancements in CNT technology, a critical challenge persists: the direct growth of vertically aligned CNTs on insulating substrates such as glass. This issue is particularly relevant for the development of transparent, large-area, and cost-effective devices. Glass substrates offer inherent advantages, including optical transparency, chemical stability, and compatibility with standard microfabrication techniques [14,15,16,17,18]. However, their low softening point poses a technical barrier to the high temperatures typically required for chemical vapor deposition (CVD), often leading to compromised CNT crystallinity and surface morphology [12]. Additionally, achieving strong adhesion and vertical alignment on inert, non-catalytic glass surfaces remains difficult, limiting the integration of CNTs into transparent substrates—especially in applications demanding precise emitter geometries, structural integrity, and reliable field emission performance [19]. Although CNT growth has been extensively studied on silicon, metals, and modified surfaces, there is a noticeable gap in research addressing the direct, patternable synthesis of vertically aligned CNTs on thermally stable glass substrates. Bridging this gap is essential for advancing CNT-based optoelectronic and vacuum microelectronic devices, where transparency, emitter precision, and thermal robustness are key design requirements.

In this study, we aim to address this gap by demonstrating the successful direct growth of patterned vertically aligned multi-walled CNTs (MWCNTs) on thermally stable glass substrates via high-temperature CVD. The primary objective of this study is to develop a fabrication strategy that enables site-specific CNT growth on glass without compromising substrate integrity. A thermally stable glass was selected to accommodate elevated synthesis temperatures, thereby preserving MWCNT crystallinity and morphology. To facilitate MWCNT growth, aluminum (Al) was deposited as a buffer layer and iron (Fe) as a catalyst layer. Photolithographic patterning was employed to define catalyst regions prior to growth, enabling controlled spatial geometry and emitter arrangement. The resulting MWCNT structures were evaluated for vertical alignment, adhesion, and geometric precision. To assess their functional performance, field emission measurements were conducted on both patterned and unpatterned MWCNT arrays. This study not only validates the feasibility of integrating MWCNTs with transparent substrates but also contributes to the broader understanding of patterning effects, substrate interactions, and emission behavior in MWCNT-based device platforms.

## 2. Materials and Methods

Corning Eagle2000 glass [20] was selected as the substrate material in this study due to its excellent thermal stability and compatibility with thin-film deposition processes. Al buffer layers of varying thicknesses were pre-deposited onto the glass substrates to serve as an intermediary layer, influencing the subsequent growth of CNTs. A 3 nm thick Fe catalyst layer was then deposited via thermal evaporation, ensuring uniform distribution of catalyst nanoparticles. To systematically investigate the effect of Al buffer layer thickness on the growth behavior of vertically aligned CNTs, five thickness variations (1, 3, 5, 7, and 9 nm) were employed in the experimental setup.

Prior to CNT synthesis, the Fe/Al multilayer structures underwent annealing at 750 °C for 60 min under a vacuum pressure of approximately 2.0 × 10^−2^ Torr. This step was crucial for facilitating the formation of Fe catalyst nanoparticles, which act as nucleation sites during CNT growth. CNT synthesis was subsequently carried out using acetylene (C_2_H_2_) gas as the carbon source within a homemade chemical vapor deposition (CVD) system. The CVD apparatus consisted of a thermal furnace with a 2.5-inch diameter quartz tube as the reaction chamber. The synthesis was conducted at 4.0 Torr for 10 min. Notably, the high softening point of the Corning Eagle2000 glass substrate (985 °C) ensured structural integrity, preventing substrate deformation during the thermal processing stages.

A comprehensive characterization of the synthesized CNTs was conducted using various analytical techniques. Scanning electron microscopy (SEM) was performed using a Hitachi S-3000H system (Tokyo, Japan) to observe surface morphologies and alignment of the CNTs. Transmission electron microscopy (TEM), carried out with an FEI TecnaiTM G2 F-20 S-TWIN instrument (FEI Company, Hillsboro, OR, USA), facilitated the examination of internal structures and graphitic quality. Additionally, selected area electron diffraction (SAED), integrated within the TEM framework, was employed as a crystallographic tool for structural analysis. Raman spectroscopy, utilizing a Renishaw in Via Raman system (Wotton-under-Edge, UK) with a 633 nm excitation source, was performed to extract detailed microstructural and compositional information, revealing key characteristics indicative of CNT quality.

To enable the application of vertically aligned CNTs in field emission devices, photolithography was employed to pattern the CNTs into well-defined structures. This patterning process mitigated the screening effect and optimized local electric field distribution, thereby enhancing field emission efficiency. The patterned hexagonal structures featured circular CNTs bundles with a diameter of 25 μm and a center-to-center spacing of 50 μm. These precisely arranged CNTs bundles not only contributed to the overall structural integrity but also showcased potential applications in advanced nanotechnology and electronic devices.

Field emission measurements were carried out using a homemade parallel-plate configuration within a high-vacuum system, operating at a base pressure of approximately 4 × 10^−7^ Torr. The CNT sample (cathode) was placed on a stainless steel stage, while the anode consisted of a stainless steel plate. The inter-electrode gap was adjustable, and a fixed distance of 150 μm was maintained during measurements. The current–voltage (I–V) characteristics and long-term emission stability of the CNT cathode were systematically evaluated. In this experiment, the applied voltage could be increased up to 1000 V using a source meter, enabling precise control and monitoring of the field emission behavior.

To further explore practical applications, a phosphor screen test was performed. The chemical composition of the green phosphor powder used in this study was BaMg_2_Al_16_O_27_:Eu, which is a europium-doped barium magnesium aluminate known for its high luminescence efficiency. Phosphor powders were uniformly sprayed onto an identical Corning Eagle2000 glass substrate, which served as the anode in the field emission setup. Under an applied electric field of 3.5 V/μm, field emission-induced luminescence was successfully demonstrated. This result underscores the potential utilization of CNTs in field emission display technologies and other optoelectronic applications.

## 3. Results and Discussion

**Figure 1:**
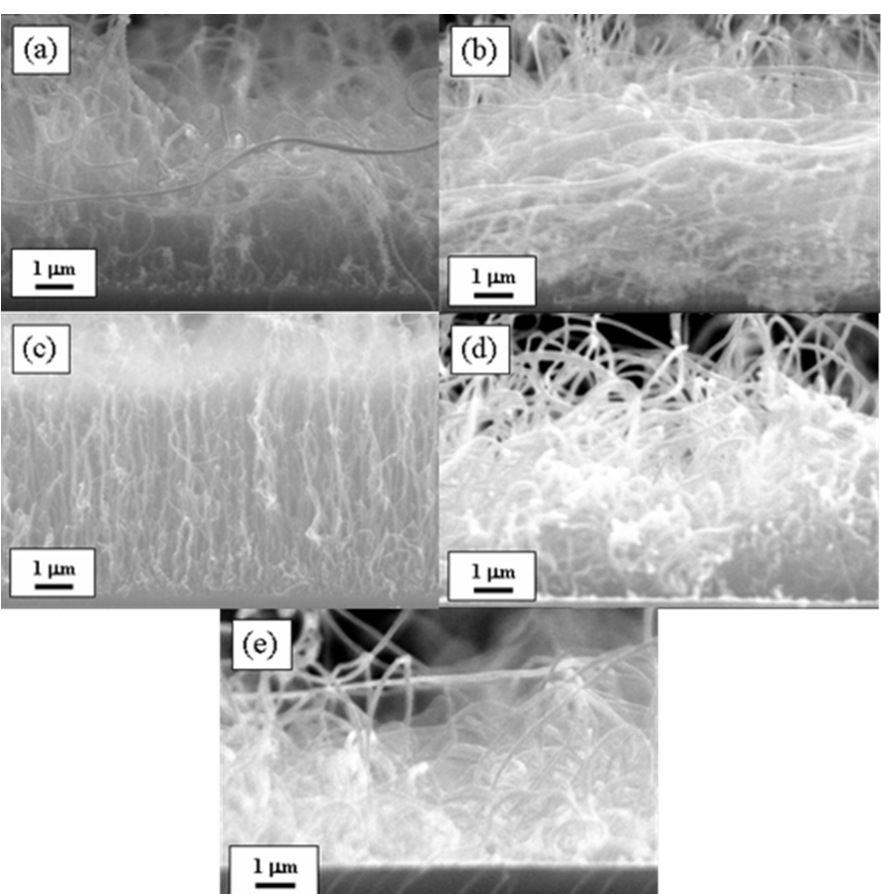
Cross-sectional SEM images of CNTs synthesized on glass substrates with a 3 nm Fe catalyst layer and Al buffer layers of different thicknesses: (**a**) 1 nm, (**b**) 3 nm, (**c**) 5 nm, (**d**) 7 nm, and (**e**) 9 nm. The images illustrate the influence of Al buffer layer thickness on CNT growth morphology.

Figure 1 shows cross-sectional SEM images of CNTs synthesized on glass substrates, each coated with Al buffer layers of varying thicknesses (1, 3, 5, 7, and 9 nm). These images clearly demonstrate that the thickness of the Al buffer layer significantly influences the resultant surface morphology of the CNTs. Specifically, for Al thicknesses of 1, 3, 7, and 9 nm (Figure 1a,b,d,e, respectively), the CNTs show a random and entangled growth pattern. In contrast, when the Al buffer layer thickness is 5 nm (Figure 1c), the CNTs exhibit a well-defined, vertically aligned structure with respect to the glass substrate surface. The vertically aligned CNTs formed under this condition reach an average length of approximately 7 μm and a number density of about 10^9^ cm^−2^. The Al buffer layer plays a pivotal role in determining the growth characteristics and alignment of CNTs. During the annealing process, the Al buffer undergoes a morphological transformation, forming nanoscale clusters [21]. These clusters subsequently act as a template for the formation of Fe catalyst nanoparticles, which are derived from a deposited Fe film. The size, number density, and spatial distribution of these Fe nanoparticles are strongly influenced by the underlying Al cluster morphology, which itself is a function of the Al buffer layer thickness. As a result, the structural configuration of the Fe nanoparticles directly governs the nucleation behavior and orientation of the growing CNTs. In this study, it was found that the most favorable condition for achieving vertical alignment was the use of an Al layer of 5 nm thickness. This specific thickness promotes the formation of appropriately sized Al clusters, which support the uniform dispersion of Fe nanoparticles necessary for vertically aligned CNT growth.

**Figure 2:**
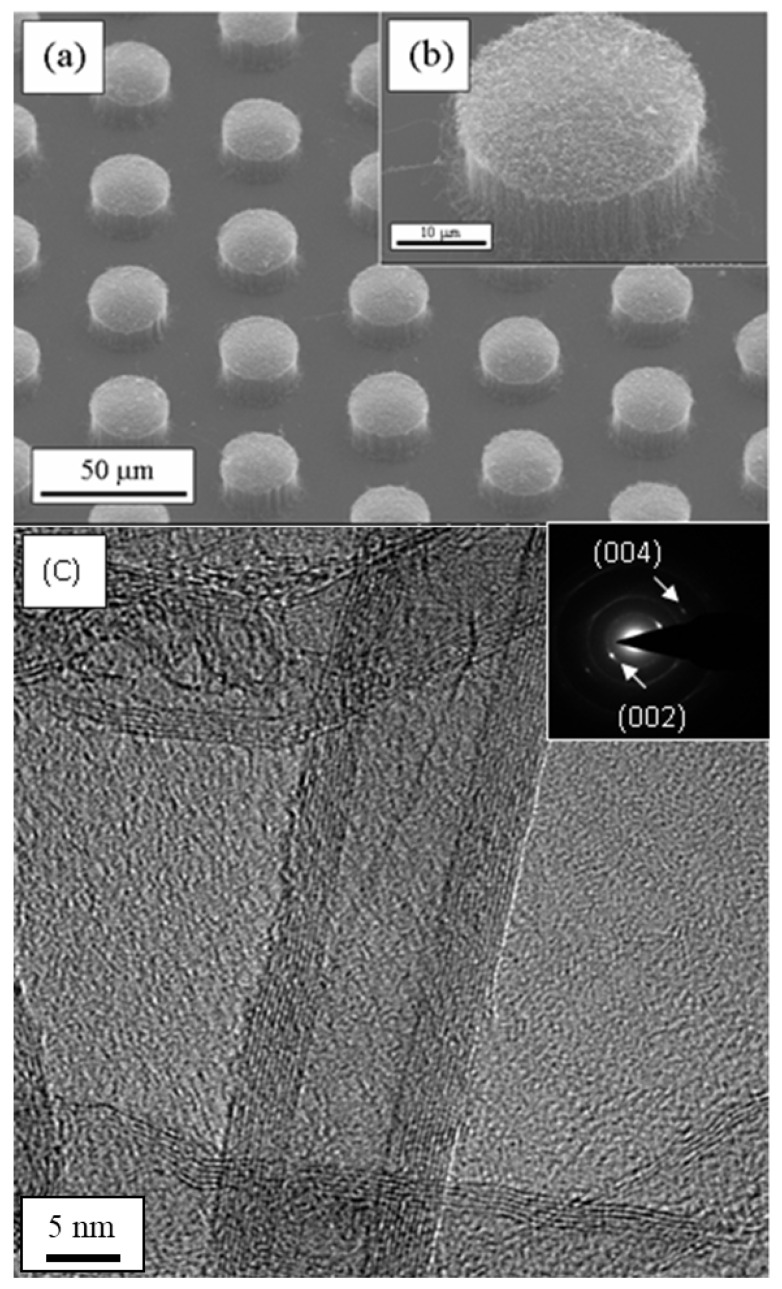
(**a**) SEM image showing vertically aligned CNT bundles grown on a glass substrate. (**b**) High-magnification SEM image of an individual CNT bundle, highlighting its vertical alignment. (**c**) TEM image of MWCNTs synthesized on glass substrates; the inset presents the SAED pattern, confirming the crystalline nature of the CNT walls.

Vertically aligned on the glass substrate, CNTs provide precise control over both height and surface morphology, significantly enhancing electron emission stability and efficiency in field emission applications. As shown in Figure 2a, CNT bundles are vertically aligned on a glass substrate in a hexagonal pattern. Each bundle has a diameter of 25 μm, with a center-to-center spacing of 50 μm. This well-structured hexagonal arrangement aligns with photolithography design principles, ensuring seamless integration with conventional microfabrication techniques. Arranging individual emitters in a discrete manner helps concentrate the electric field, further improving field emission performance [22]. Therefore, photolithography-based patterning of CNTs can also be successfully implemented on this glass substrate.

In Figure 2b, a magnified image of a single vertically aligned CNT bundle reveals a high number density of CNTs within each bundle, which is about 10^9^ cm^−2^. This high number density arrangement allows the CNTs bundle to function as a single field emitter, significantly boosting its electron emission potential. Such dense configurations ensure that the CNT bundles are well-suited for applications requiring high-performance field emitters, demonstrating their potential for both enhanced stability and efficiency in a range of electronic and energy applications [23].

The growth of vertically aligned CNTs in this study is primarily governed by the structure of the catalyst layer, which is strongly influenced by the thickness of the underlying Al buffer layer. During annealing, the Al buffer undergoes a morphological transformation into nanoscale clusters, which serve as templates for the formation of Fe catalyst nanoparticles. An Al thickness of 5 nm produces optimally sized and uniformly distributed Fe nanoparticles, enabling consistent CNT nucleation and vertical growth. The high number density of catalyst nanoparticle, approximately 10^9^ cm^−2^, ensures dense CNT arrays. Furthermore, at such high number densities, van der Waals interactions between adjacent CNTs generate mutual repulsion, driving them to self-assemble in an upright orientation. This combination of catalyst size control, uniform dispersion, and intertube interactions results in well-aligned CNT arrays with improved structural uniformity and properties suitable for high-performance applications, which is consistent with previous reports on the critical roles of buffer layer engineering, catalyst nanoparticle stability, and self-assembly effects in vertically aligned CNT growth [24,25,26].

Figure 2c shows a TEM image of CNTs grown on a glass substrate. The image reveals that the inner structure of the vertically aligned CNTs is hollow, confirming its characteristic morphology. The CNTs exhibited a multi-walled structure, with an outer diameter of approximately 17 nm. This multi-walled configuration contributes to the enhanced mechanical strength and stability of the CNTs, making them ideal for various applications in nanotechnology and electronics. The inset in Figure 2c shows the SAED pattern of the CNTs. The diffraction pattern confirms the good crystallinity of the vertically aligned CNTs, as evidenced by the well-defined graphite (002) and (004) diffraction spots [27,28]. These clear spots indicate that the CNTs exhibit high-quality crystalline alignment, which is crucial for optimizing their electronic and mechanical properties.

**Figure 3:**
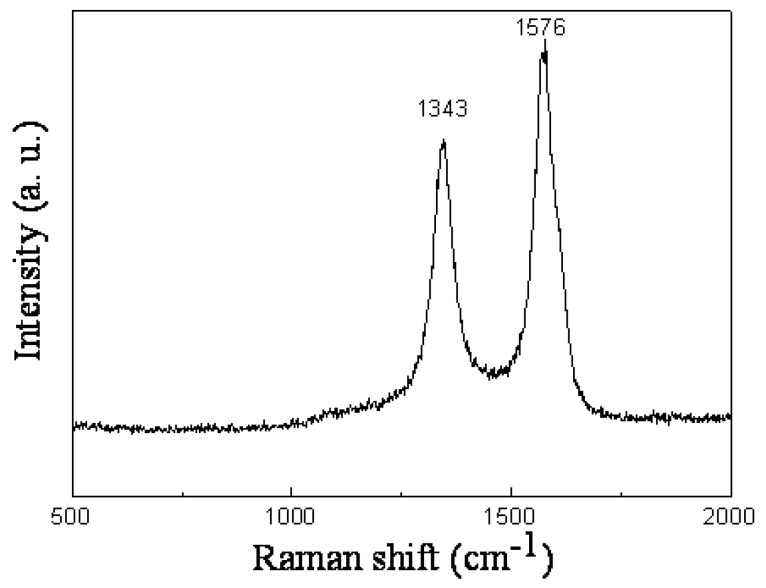
Raman spectrum of vertically aligned MWCNTs, showing characteristic D and G bands indicative of graphitic structure and defect levels.

Figure 3 shows the Raman spectrum of the vertically aligned MWCNTs, highlighting two prominent resonance peaks. The D-band, located at 1343 cm^−1^, and the G-band, located at 1576 cm^−1^, are clearly observable. The D-band is typically associated with the presence of disorder in the carbon structure, indicating some level of defects or irregularities within the CNTs. On the other hand, the G-band corresponds to the stretching vibration of sp^2^-hybridized carbon atoms, and is a characteristic feature of crystalline graphitic carbon, which in this case reflects the high-quality alignment and crystalline nature of the vertically aligned CNTs [29,30].

**Figure 4:**
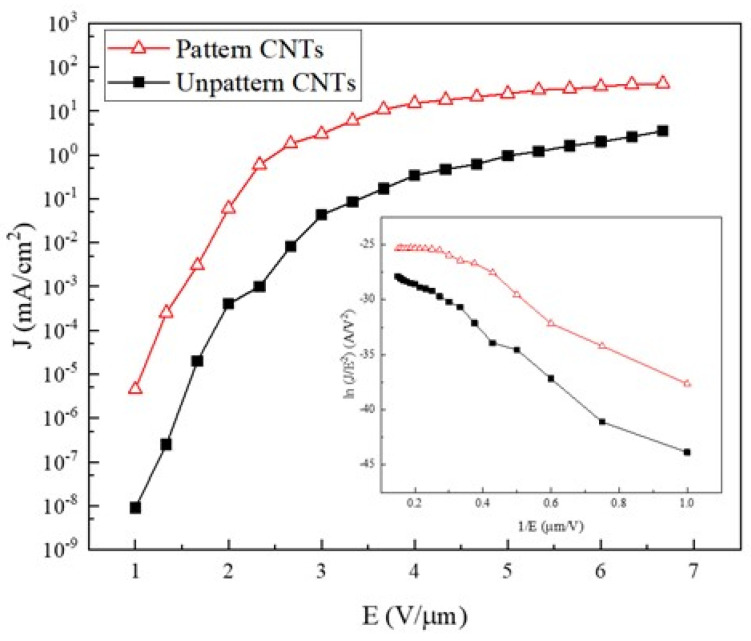
Comparison of field emission characteristics between unpatterned vertically aligned MWCNTs and patterned arrays of vertically aligned MWCNT bundles.

In this application, we conducted field emission measurements using vertically aligned MWCNTs grown on a glass substrate, with surface morphologies classified as either unpatterned or patterned. Figure 4 shows the current density (J) versus electric field (E) characteristics of both types: unpatterned vertically aligned CNTs and patterned vertically aligned CNTs bundles. The threshold electric field (Eth) was defined as the field corresponding to a current density of 10^−1^ mA/cm^2^. The Eth values for the unpatterned and patterned CNTs structures were approximately 3.3 V/μm and 2.0 V/μm, respectively. Notably, the field emission performance of the patterned vertically aligned CNTs bundles was clearly superior to that of the unpatterned CNTs. This improvement is attributed to the consistent spacing between adjacent CNTs bundles arranged in a hexagonal configuration.

The inset in Figure 4 shows the Fowler–Nordheim (FN) plots, which appear nearly linear, indicating that the emission behavior follows the FN relationship [31]. The FN equation is given by the following: J = A(β^2^E^2^/Φ)∙exp(−BΦ^3/2^/βE) where A (1.54 × 10^−6^ AeV/V^2^) and B (6.8 × 10^3^ eV^−3/2^ V/μm) are constants. J is the field emission current density, β is the local field enhancement factor, E is the applied electric field (V/μm), and Φ is the work function (4.8 eV for CNTs) [32]. The β values for the unpatterned and patterned vertically aligned CNTs structures were approximately 3882 and 4188, respectively.

**Figure 5:**
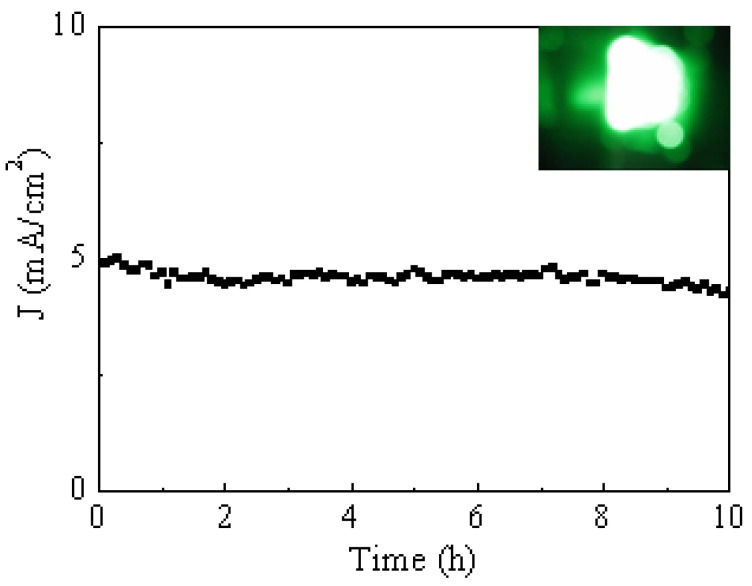
Time dependence of the field emission current density at 3.5 mA/cm^2^. The inset shows the fluorescent screen used to monitor the field emission, which is displaying a uniform emission pattern.

Figure 5 illustrates the stability of the field emission current density measured over a continuous period of 10 h. The inset image displays the fluorescent screen illuminated by MWCNTs bundles under an applied electric field of 3.5 V/μm, clearly demonstrating uniform emission sites. The consistent current output and uniformity of the emission pattern highlight the excellent operational stability of the vertically aligned MWCNTs bundles. These results strongly suggest that such structures are highly promising candidates for practical integration into field emission devices, offering both reliability and efficiency. Table 1 shows a comparison of the field emission properties of different CNT structures between this study and other research groups [33,34,35]. It can be observed that vertically aligned CNTs exhibit a higher current density than randomly grown CNTs. The results of this study demonstrate the feasibility of growing vertically aligned MWCNTs on glass substrates.

## 4. Conclusions

In this study, we successfully demonstrated the direct growth of vertically aligned MWCNTs on Eagle2000 glass substrates using CVD. The Al buffer layer thickness was identified as a key factor influencing CNT morphology, with an optimal 5 nm layer enabling the formation of uniformly dispersed Fe nanoparticles that promote vertical alignment. In addition to direct integration on transparent glass, we achieved precise photolithographic patterning of CNTs into hexagonally arranged bundles. This study establishes the successful demonstration of patterned, vertically aligned MWCNTs directly grown on Eagle2000 glass at elevated temperatures, overcoming persistent challenges of adhesion, alignment, and thermal stability on insulating, transparent substrates. Moreover, the patterned MWCNT bundle arrays exhibited enhanced field emission performance, characterized by remarkable current stability and highly uniform phosphor luminescence, underscoring their suitability for transparent field emission displays and advanced optoelectronic devices.

## Figures and Tables

**Table 1:** Comparison of the field emission characteristics of different CNT structures.

Pattern Geometry	Threshold Field (V/μm)	Current Density (mA/cm^2^)	β	Reference
Vertically well-aligned CNTs	-	80 at 3 V/μm	-	[33]
CNT carpet	0.48 at 1 mA/cm^2^	2.13 at 0.97 V/μm	19,141	[34]
Vertically well-aligned CNTs	1.6 at 10 mA/cm^2^	166 at 2.35 V/μm	-	[35]
Random growth CNTs	3.3 at 0.1 mA/cm^2^	3.5 at 6.67 V/μm	3882	This study.
Vertically aligned hexagonal CNTs	2.0 at 0.1 mA/cm^2^	42 at 6.67 V/μm	4188	This study.

## Data Availability

The original contributions presented in this study are included in the article. Further inquiries can be directed to the corresponding author.

## Abbreviations

The following abbreviations are used in this manuscript:

CNTs	Carbon nanotubes
CVD	Chemical vapor deposition
SEM	Scanning electron microscopy
TEM	Transmission electron microscopy
SAED	Selected area electron diffraction
FN	Fowler–Nordheim
